# N-terminal moiety of Antimicrobial peptide Ltc1-k increases its toxicity for eukaryotic cells 

**Published:** 2011

**Authors:** O.V. Samsonova, K.S. Kudryashova, A.V. Feofanov

**Affiliations:** Biological Faculty, Lomonosov Moscow State University; Shemyakin and Ovchinnikov Institute of Bioorganic Chemistry, Russian Academy of Sciences

**Keywords:** antimicrobial peptides, latarcin, haemolytic activity, circular dichroism, fluorescent confocal microscopy

## Abstract

The antimicrobial peptide Ltc1-K and its derivates without one, two, then three N-terminal amino acid residues were studied based on the hypothesis (backed by some experimental data) that the hydrophobic N-terminal moiety of linear cationic antimicrobial peptides defines their haemolytic activity. It was discovered that the excision of three N-terminal amino acid residues considerably decreases the peptide’s toxicity for eukaryotic cells and simultaneously increases the selectivity of antibacterial activity for some bacteria species. Studies performed with the model membrane systems and human erythrocytes revealed that the main reason for the observed effect is a multifold decrease in the peptide’s affinity to an eukaryotic cellular membrane enriched with zwitterionic phospholipids.

## INTRODUCTIONS 

Antimicrobial peptides (AMP) are natural substances that vary in structure and biological functions. They possess in common the capability of inhibiting the vital activity of pathogenic microorganisms. Endogenic AMPs are considered to be one of the key factors (and simultaneously an evolutionarily ancient one) of the innate immunity of multicellular organisms, which enables them to be regarded as prototypes for a new generation of antibiotics [1–3]. The search for new AMPs and the detailed study of the existing ones is stimulated by the development of resistance to conventional antibiotics in microorganisms and by the capability of many AMPs to overcome this resistance.

A new group of AMPs consisting of seven short cationic linear peptides – latarcins – have recently been isolated from the poison of the Central Asian spider *Lachesana tarabaevi * and characterized [4]. Latarcins Ltc1, Ltc2, and Ltc5, which are similar to a number of other AMPs, along with moderate haemolytic activity, display high antibacterial activity [4]. AMPs are regarded as an alternative to existing antibiotics: therefore, their cytotoxicity with respect to human cells is undesirable. The structural determinants of the cytotoxicity of AMPs have been revealed both from the research and practical perspectives. One of such structural elements was identified in linear α-helix AMPs [5]. It was ascertained that the hydrophobic N-terminal fragment enhances the haemolytic activity of these peptides, based on the structural and functional analysis of a large number of α-helix AMPs. Using the example of latarcins Ltc2a and Ltc5, it was demonstrated that the activity of AMPs with respect to eukaryotic cells can be reduced via the removal of their N-terminal fragment or via point amino acid replacements that decrease their hydrophobic potential [5].

We follow this direction and present the results of an investigation into the properties of AMP Ltc1-K ( *Table 1* ), which corresponds to the non-processed form of latarcin Ltc1 with an additional lysine residue on the C-terminus. In terms of haemolytic activity and bactericide effect with respect to *Escherichia coli* and *Bacillus subtilis* , Ltc1-K does not differ from mature Ltc1 [6]. It was demonstrated by NMR that when Ltc1-K binds to sodium dodecylsulfate micelles (the simplest membrane-imitating model), the N-terminal fragment of the α-helix peptide is embedded into the hydrophobic region of a micelle [7]. Taking into account the NMR data [7] and structural and functional analysis [5], we assumed that the N-terminal fragment enhances the binding of Ltc1-K to the membranes consisting of zwitterionic lipids and determines the cytotoxic activity of a peptide. Three Ltc1-K analogues shortened from the N-terminus by one, two, and then three amino acid residues ( *Table 1* ) were synthesized in order to verify this hypothesis. The comparative analysis of their structures, activity, and features of haemolytic action was performed.

## EXPERIMENTAL 

**Reagents**

**Table 1:** Amino acid sequences of Ltc1-K and its derivatives

Peptide	Amino acid sequence
Ltc1-K	SMWSGMWRRKLKKLRNALKKKLKGEK
(-1)Ltc1-K	MWSGMWRRKLKKLRNALKKKLKGEK
(-2)Ltc1-K	WSGMWRRKLKKLRNALKKKLKGEK
(-3)Ltc1-K	SGMWRRKLKKLRNALKKKLKGEK

The following reagents were used in this study: 1,2-dioleoyl- *sn* -glycero-3-phosphocholine (DOPC) and 1-myristoyl-2-hydroxy- *sn* -glycero-3-phosphocholine (LMPC) (Avanti Polar Lipids, United States); Mueller – Hinton broth, carboxyfluorescein (CF), fluorescein-labelled dextrans with molecular weight 70 and 500 kDa (FD70 and FD500), fluorescent dyes FM1-43, propidium iodide, and Hoechst 33342 (Sigma Aldrich, United States); culture medium Roswell Park Memorial Institute 1640 (RPMI-1640) purchased from PanEco (Russia); and heparin (Sintez, Russia). The remaining reagents were of special purity grade.

The peptides ( *Table 1* ) were synthesized by solid phase synthesis in the Peptide Synthesis Laboratory of the Institute of Bioorganic Chemistry of the Russian Academy of Sciences (Moscow) (team leader N.S. Egorova) and characterized by HPLC and mass spectrometry (MALDI-MS) as described earlier [4, 6]. Peptide concentrations in aqueous solutions were determined on the basis of UV absorption using a SF-256 spectrophotometer (LOMO, Russia) and the molar extinction coefficients 5500 M ^-1^ cm ^-1 ^ for (-3)Ltc1-K and 11000 M ^-1^ cm ^-1^ for Ltc1-K, (-1)Ltc1-K, and (-2)Ltc1-K at a wavelength of 280 nm.

**Recording and analysis of the circular dichroism (CD) spectra**

The CD spectra of the peptides were recorded in a phosphate buffered saline (110 mM NaCl, 50 mM NaH _2_ PO _4_ ,pH 7.4) and in LMPC micelles (30 mM with respect to lipid) in a phosphate-buffered saline at a peptide concentration of 40 µM, using a Jasco J-810 spectropolarimeter (Jasco, Japan). In order to attain an equilibrium distribution of the peptide over the micelles, LMPC-containing samples were pre-incubated for 30 min at a temperature of 25°С. The spectra were recorded within a range from 190 to 250 nm with a 0.2 nm increment (the slit spectral width being 1 nm). A sample was placed into a cell with a detachable window (Hellma, Germany) with an optical path length of 0.01 cm. The secondary structures of the peptides were calculated on the basis of the CD spectra using CONTILL software [8]. The calculation results were averaged on the basis of two independent experiments.

**Preparation of erythrocyte suspension**

The donor’s capillary blood (100–150 µl) was mixed with a RPMI-1640 medium containing heparin (10 u/ml). The cells were precipitated by centrifugation for 2 min at 300 g; the precipitate was re-suspended in a heparin-free medium until a final erythrocyte concentration of 2 × 10 ^7^  CFU/ml was attained. All experiments were carried out during 2–3 h after blood sampling.

**Analysis of haemolytic activity**

The erythrocyte suspension was mixed at an equivolumetric ratio with a peptide that was preliminarily diluted by RPMI-1640 to the desired concentration, in order to ascertain the haemolytic activity of peptides; the mixture was then incubated for 1 h (37°С) under constant stirring (140 rpm). Fetal bovine serum (8%) was either added or not added to the mixture. The cells were then precipitated by centrifugation (1200 g, 5 min), and supernatant was taken. The haemoglobin release from erythrocytes was estimated on the basis of the optical density of supernatant at a wavelength of 414 nm, measured on a photometric plate reader Uniplan (PICON Russia). The haemolysis degree was calculated using the following formula: 

HC _%_ = (OD _e _ – ОD _0_ )/(ОD _100_ – ОD _0_ ) × 100%,(1)

where OD _e_ , OD _0_ , and OD _100_ are the optical densities (414 nm) of supernatants obtained from the sample under study, from the control cells and the totally lysed cells, respectively. For total lysis of erythrocytes, the RPMI-1640 medium was substituted for deionised water. The results of two independent experiments, with measurements repeated thrice in each of them, were averaged. The equivalent amount of a peptide-free medium was added to the control cells. The concentration dependence of haemolysis was approximated by the sigmoid dependence equation; the peptide concentration that caused a 50% release of haemoglobin from erythrocytes (HC _50_ ) was determined.

**Analysis of cytotoxicity**

Human erythromyeloid leukemia К562 cells were cultured (5% CO _2_ , 37°C) in a RPMI-1640 medium with 8% fetal bovine serum and L-glutamine (2 mM). The cytotoxicity of peptides was determined as described earlier [9]. The cells were incubated for 3 h (5% CO _2_ , 37 ^o^ C) in a medium containing the peptides under study in serial dilutions (from 0.1 to 100 µM). Then, propidium iodide (10 µM) and Hоеchst 33342 (10 µM) were introduced into the medium; after 10 min, the state of the cells was analyzed using an Axiovert 200M fluorescence microscope (Carl Zeiss, Germany). The cells stained only with Hoechst 33342 were considered to be living, whereas the cells stained with Hoechst 33342 and propidiun iodide were regarded as dead. At least 500–1,000 cells were analyzed for each peptide concentration. The results of two independent experiments were averaged. The equivalent amount of the peptide-free medium was added to the control cells. The concentration dependence of cytotoxicity was approximated by the sigmoid curve, and the peptide concentration causing the death of 50% of cells (EC _50_ ) was determined.

**Analysis of antibacterial activity**

Cells from *E. coli* C600, *Micrococcus luteus* Ac-2230 VKM, and *B. subtilis* B-501 VKM were cultured in a Mueller–Hinton broth (37°C). The anti-bacterial activity of the peptides was measured using the method of serial dilution in a liquid medium as described earlier [9]. The bacterial suspension (10 ^5^  CFU/ml) was incubated in the Mueller–Hinton broth in the presence of serial dilutions of peptides for 24 h at 37°C. The absence of bacterial reproduction served as the criterion of peptide activity; the reproduction was detected by comparing the optical density (595 nm) of the bacterial culture in the presence of the peptide with that in the control using the photometric plate reader Uniplan (PICON Russia). The results of two independent experiments, the measurements being repeated thrice in each of them, were averaged. The equivalent amount of the peptide-free medium was added to the control cells. The activity was characterized by the minimum inhibitory concentration (MIC) value.

**Preparation of large monolamellar liposomes (LML)**

A weighed portion of DOPC was hydrated in a phosphate buffered saline solution(110 mM NaCl, 50 mM NaH _2_ PO _4_ , pH 7.4) for 2 h at 25°С upon vigorous shaking. The suspension of multilamellar liposomes with DOPC at a concentration of 10 mM was obtained by the freeze–thaw method in liquid nitrogen (10 cycles). An LML with a diameter of 100 nm was prepared by extrusion of the multilamellar liposome suspension through a polycarbonate filter with a 100-nm-pore diameter (Whatman, Great Britain) according to the recommendations of the manufacturer of the mini-extruder (Avanti Polar Lipids, United States).

**Studying the interaction between peptides and DOPC liposomes**

The level of binding to the lipid bilayer was assessed on the basis of the short-wavelength shift of the fluorescence spectrum of Trp residues within the peptides as the side chain of Trp transferred from the polar environment into a hydrophobic one. The fluorescence spectra were recorded on an LS 55 spectrofluorimeter (Perkin Elmer, Great Britain) at 25°С. Fluorescence was excited at 270 nm, and the emission spectrum was registered within a range from 300 to 500 nm with a measurement increment of 0.5 nm. In order to reduce the distortions of the spectra due to light scattering on liposomes, a quartz cell with a 10 × 2 mm cross-section was used when recording the fluorescence of a thin layer of the sample (2 mm). 

The samples contained 2 µM of peptide and 0.1–0.5 mM of DOPC in the form of LML in the phosphate buffered saline. The recorded spectra were analyzed in LabSpec2.0 software (Dilor, France). The mathematical procedure of representing the experimental spectrum as a sum of two spectra (a peptide in the aqueous environment and a peptide totally bound to lipids) was used with the corresponding weight coefficients in order to calculate the ratio between the bound and unbound forms of the peptide. The suspension of zwitterionic LMPC micelles (20 mM lipid) was used to measure the spectrum of the peptide that was completely bound to the lipids. The complete binding of peptides to the LMPC micelles was confirmed by the CD method based on the dependence of formation of the α-helix conformation of peptides on micelle concentration. 

The dissociation constant ( *K*_d_ ) was determined according to the earlier described procedure [10], based on the data averaged in two independent experiments, using the following formula:

*С*_M_ / *L* = 1/ *K*_d_ × С _buf,_ (2)

where *С*_buf_ is the concentration of the unbound peptide in the solution and *С*_M_ / *L* is the concentration of the membrane-bound peptide reduced to lipid concentration.

It should be noted that at a DOPC concentration higher than 1 mM, the intensity of the fluorescence recorded decreases due to light scattering, which results in the underestimation of the C _M _ and C _buf_ values under determination. However, this fact has no effect on the calculated ratio *С*_M_ / *С*_buf_ , which is used to determine *K*_d_ .

**Microscopic studies**

When studying the peptide-induced morphological changes in erythrocytes in the RPMI-1640 medium, equal volumes of the erythrocyte solution (6 × 10 ^6^  CFU/ml) and peptide solution were mixed, ensuring equi-effective concentrations of Ltc1-K and (-3)Ltc1-K (the final concentration in the sample was 4 µM Ltc1-K or 30 µM (-3)Ltc1-K); CF (20 µM) was also added. The sample was placed into a 12-well chamber flexiPERM (Perbio, Belgium) with a 0.17-mm-thick glass bottom, centrifugated for 1 min at 250 rpm to deposit the cells onto the glass, and transferred under a microscope for analysis.

The erythrocytes, at a concentration of 10 ^7^  CFU/ml, were incubated in the presence of equi-effective concentrations of Ltc1-K (2 µM) or (-3)Ltc1-K (15 µM) in the RPMI-1640 medium for 20 min at 37°С in order to obtain ghosts. Membrane permeability markers (CF, FD70 or FD500) at a concentration of 20 µM and FM1-43 (0.9 µM) staining the plasma membrane were then added. The incubation of the samples was continued for an additional 1 h at 37°С. The samples were then analyzed analytically.

An LSM 510 Meta laser scanning confocal microscope (Carl Zeiss, Germany) with a C-Apochromat 63×/1.2 W objective was utilized. The measurements were performed with a lateral resolution of 0.3 µM and an axial resolution of 0.6 µM. The fluorophores were excited by an Ar ^+^ laser with wavelengths of 458 nm (FM1-43) and 488 nm (CF, FD70, and FD500). The fluorescence was recorded by isolating the desired spectral range using a band-pass filter at 505–550 nm (CF, FD70, and FD500) and a long-wave barrier filter with the boundary set at 585 nm (FM1-43). The transmitted light images of cells were recorded simultaneously with the fluorescence images.

When studying the dynamics of the processes, 100 repeated measurements were performed with an increment of 2 s. 

The images were processed using the ImageJ software (National Institute of Health, United States). The extent of influx ( *A* ) of the membrane permeability markers (CF, FD70, FD500) inside an erythrocyte (%) was calculated using the following formula:

*A* = ( *F*_in _ – *F*_0_ )/( *F*_out_ – *F*_0_ ) × 100,(3)

where *F*_in_ is the average fluorescence intensity inside the ghost, *F*_out_ is the average fluorescence intensity of the medium around the cells, and *F*_0_ is the background signal inside the intact erythrocytes. The extent of influx was averaged with respect to 100–150 cells.

## RESULTS and DISCUSSION 

**Biological activities of peptides**

The abilities of latarcin Ltc1-K and its derivatives ( *Table 1* ) to lyse erythrocytes and human erythromyeloid leukemia cells and inhibit *in vitro* bacterial growth ( *Table 2* ) were compared in order to assess the effect of the N-terminal fragment on haemolytic, cytotoxic, and antibacterial activities.

The excision of one to three N-terminal amino acid residues had no effect on the capability of the peptides to inhibit the growth of the gram-positive bacteria *B. subtilis* . The activity with respect to gram-negative *E. coli * and gram-positive *M. luteus * decreased considerably only for (-3)Ltc1-K. No interrelation between the change in the range of activity of Ltc1-K upon excision of the N-terminal fragment and the morphological or tinctorial properties of the bacteria was established: the activity decreased not only with respect to gram-negative * E. coli* bacilli, but also for gram-positive cocci *M. luteus* , which have no lipopolysaccharide core. Meanwhile, the sensitivity of the gram-positive bacilli *B. subtilis * remained at the same level. It can be assumed that the decrease in activity observed is to a large extent associated with the features of interaction between the peptides and the plasma membrane of certain types of bacteria.

Peptide-mediated permeabilization of the bacterial membrane is considered to be the most probable mechanism of antibacterial effect of Ltc1-K and its analogues, as well as that of other cationic AMPs. The data on the ability of the peptides studied to induce defects in the erythrocyte membrane indirectly attest to this fact. Permeabilization of the bacterial membrane can occur either by formation of toroidal lipid-peptide pores or by the so-called “carpet” mechanism, which causes detergent-like damage to the membrane [11]. In the former case, the excision of the hydrophobic N-terminal fragment is more likely to reduce the ability of the peptide to weave into the hydrophobic membrane region and initiate the formation of lipid-peptide pores. In the latter case, the excision of the N-terminal fragment will have a smaller effect on the efficacy of membrane damage. In both cases, the initial binding of a peptide to the membrane is determined by electrostatic interactions between the cationic amino acid residues and anionic lipids, in which the bacterial membrane is enriched. In addition, the excision of the N-terminal fragment of Ltc1-K does not affect the electrostatic interactions.

Specific mechanisms of defect formation in the membrane of different types of bacteria may depend on the composition and rigidity of the membrane. Hence, the activity of (-3)Ltc1-K with respect to *E. coli * and *M. luteus* (lower than that of Ltc1-K) may imply the permeabilization of the membrane of these bacteria via the mechanism of formation of toroidal lipid-peptide pores, whereas a detergent-like damage to the membrane should be assumed in the case of *B. subtilis* . The experimental verification of this hypothesis requires a specific long-term study and lies beyond the scope of the present article.

**Table 2:** Characteristics of interaction of Ltc1-K and its derivatives with erythrocytes, K562 cells, bacteria and DOPC-liposomes

Peptide	Eukaryotic cells		Bacteria			DOPC-liposomes
	Erythrocytes HC_50_, µM	K562 EC_50_, µM	E. coli MIC, µM	B. subtilis MIC, µM	M. luteus MIC, µM	*K*_d_, mM
Ltc1-K	1.1 ± 0.1	7.1 ± 0.4	1.3± 0.3	0.7 ± 0.2	1.1 ± 0.3	1.4 ± 0.5
(-1)Ltc1-K	0.8 ± 0.1	4.9 ± 0.2	2.6± 0.6	0.7 ± 0.2	2.7 ± 0.6	1.4 ± 0.7
(-2)Ltc1-K	1.3 ± 0.1	11 ± 1	2.6± 0.6	0.7 ± 0.2	2.9 ± 0.6	1.7 ± 0.6
(-3)Ltc1-K	8.0 ± 2.0	39 ± 5	6.5± 1.6	0.7 ± 0.2	5.6 ± 0.9	15 ± 3

Note. HC _50_ – peptide concentration resulting in haemolysis of 50% of erythrocytes. EC _50 _ – peptide concentration resulting in death of 50% of cells. MIC – the minimum inhibitory concentration inhibiting the growth of a microorganism in a liquid nutrient medium. *K*_d_ **– ** dissociation constant of peptide complexes with DOPC-liposomes.

**Fig. 1:**
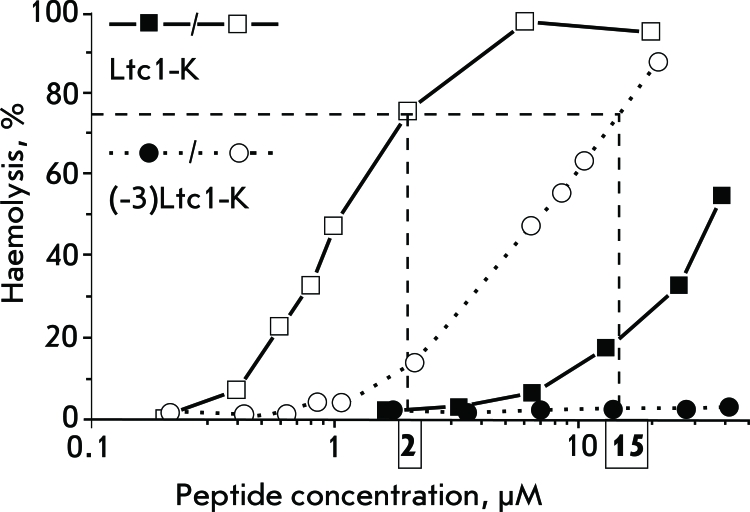
Concentration dependence of the haemolytic effects of Ltc1-K and (-3)Ltc1-K. Open symbols correspond to erythrocyte treatment with peptides in a serum-free medium, closed symbols correspond to erythrocyte treatment with peptides in a medium complemented with a 8% fetal bovine serum. Each line is a best fit of the experimental data with a Hill function. Vertical dashed lines indicate the equi-effective Ltc1-K and (-3)Ltc1-K concentrations that were used in subsequent experiments.

No considerable differences in the haemolytic activity of Ltc1-K, (-1)Ltc1-K, and (-2)Ltc1-K has been detected, whereas the activity of (-3)Ltc1-K decreased by a factor of 7 ( *Table 2* ). A similar regularity was revealed during the analysis of the cytotoxicity of peptides with respect to K562 cells ( *Table 2* ). Let us note that, for convenience, haemolysis of erythrocytes by the peptides was compared in a medium containing no serum; its presence (same as the presence of blood plasma) protects erythrocytes and reduces the haemolytic activity of peptides down to moderate values. Thus, in the presence of 8% of fetal bovine serum, the HC _50_ values for Ltc1-K, (-1)Ltc1-K, and (-2)Ltc1-K are 28, 67, and 58 µM, respectively. Attempts at measuring HC _50 _ for (-3)Ltc1-K over the available concentration range were unsuccessful ( *Fig. 1* ).

By comparison, HC _50_ of mellitin from the poison of *Apis mellifera* , which belongs to the highly active haemolytic peptides, in the presence of 8% of serum is 0.3 µM (the data are not given), whereas HC _50_ of the earlier studied latarcins Ltc2a and Ltc5 are 6 and 12 µM, respectively [5]. The cytotoxicity (EC _50_ ) of Ltc2a and Ltc5 for K562 cells is 3 and 12 µM [5]. The haemolytic activity of Ltc1-K is noticeably less than that of Ltc2a, Ltc5, and mellitin, but it is comparable with the activity of other latarcins with respect to K562 cells. The excision of the N-terminal fragment is accompanied by a multifold decrease in the cytotoxicity and haemolytic activity of Ltc2a, Ltc5 [5], and Ltc1-K. Thus, the resulting data ( *Table 2* ) lend support to the earlier conclusions [5] that the haemolytic and cytotoxic activities of α-helix AMPs are enhanced by the hydrophobic potential of the N-terminal fragment. In the case of Ltc1-K, the considerable decrease in membrane-lytic activity is attained after the N-terminal hydrophobic residue Trp3 is removed.

**Fig. 2:**
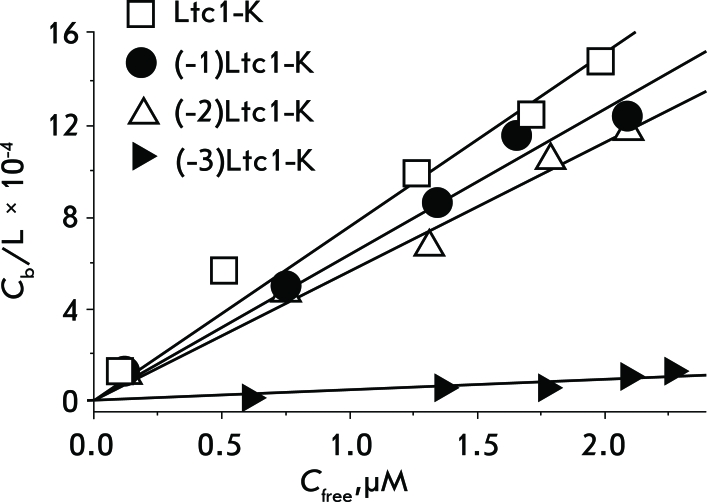
Association isotherms for the binding of Ltc1-K, (-1)Ltc1-K, (-2)Ltc1-K, and (-3)Ltc1-K to DOPC-LML. Solid lines are a least squares approximation of experimental data with a linear function defined by equation (2).

The features of interaction of Ltc1-K and its derivatives with the erythrocytes and lipid systems (DOPC-liposomes, LMPC micelles) that simulate the membrane of eukaryotic cells were studied in order to establish the reasons for the reduction in toxicity. 

**Comparative analysis of peptide affinity to DOPC-liposomes**

Assuming that the toxicity with respect to eukaryotic cells depends on the peptide affinity towards the zwitterinoic lipids dominant within the plasma membrane, we measured the dissociation constants ( *К*_d_ ) of the complexes of Ltc1-K and its derivatives with DOPC-liposomes.

The results of the analysis of peptide binding to liposomes are represented in *Fig. 2* . As can be seen, the selected mathematical model adequately describes the resulting binding isotherms. The *К*_d _ values for the peptides Ltc1-K, (-1)Ltc1-K, and (-2)Ltc1-K determined on the basis of isotherms are equal, whereas the *К*_d_ value of (-3)Ltc1-K increases by a factor of 10 ( *Table 2* ), pointing to a considerable reduction in the affinity of (-3)Ltc1-K towards the lipid bilayer.

The estimation of the thermodynamic contribution, Δ *G* ^0^_N-term_ , of the N-terminal peptide fragment to the binding with membrane, performed using the formula

Δ *G* ^0^_N-term_ ~ RT ln ( *K*_d(Ltc1-K)_ / *K*_d__((-3)Ltc1-K)_ ),(4)

demonstrates that the free energies of interaction with the bilayer in Ltc1-K and (-3)Ltc1-K differ by 1.4 kcal/mol; i.e., the change in the free energy of interaction is ~20%. The major contribution to the free energy of interaction between the N-terminal fragment and the lipids is made by the Trp3 residue; this interaction seems to be of mostly hydrophobic character. The fact that the value Δ *G* ^0^_N-term_ is close to – 1.95 kcal/mol (the value predicted by the White–Whimley method based on the empirical scale of hydrophobicity of amino acid residues [12]) lends support to this hypothesis.

Thus, the excision of the N-terminal fragment in Ltc-K results in an abrupt decrease both in the haemolytic activity and cytotoxicity, and the *K*_d_ value, supporting the fact that peptide affinity towards the zwitterionic membrane is indeed of great significance for the development of the haemolysis and cytotoxic effects.

**Effect of the N-terminal fragment of Ltc1-K on the secondary structure of peptide in peptide–lipid complexes**

Peptide conformation is another factor that may have an effect on the activity of peptides. We compared the secondary structure of Ltc1-K and its derivatives by analyzing the CD spectra of peptides in the phosphate buffered solution and in the lipid system – micelles of zwitterionic LMPC, which is used for simulating the surface of the eukaryotic cell membrane [13]. LMPC forms a 3D hydrophobic phase that is comparable in terms of its diameter with the thickness of the hydrophobic region of the biological membrane [14]. Moreover, LMPC does not have its own spectrum in the CD region of polypeptides and is characterized by a low level of light scattering at a high concentration of the phospholipid component, which provides the possibility to attain a high excess of lipid as compared with the peptide and ensures complete binding of peptide to micelles.

**Fig. 3:**
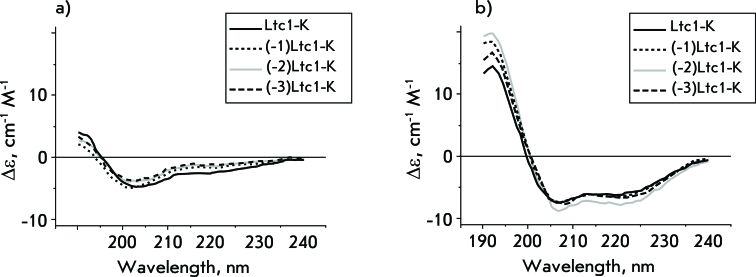
Circular dichroism (CD) spectra of Ltc1-K and its derivates in phosphate buffered saline (a) and LMPS micelles (b).

We found out that all the peptides under study in the buffer have a predominately disordered structure ( *Fig. 3* ) with an equally low content of elements with α-helix conformations ( *Table 3* ). Peptides undergo considerable conformational changes ( *Fig. 3* ) when bound to zwitterionic micelles: the α-helix becomes the predominant structural element, with 15–17 amino acid residues involved in its formation ( *Table 3* ). The results obtained correlate with the data on the high potential of helix formation in Ltc1-K in 50% of trifluoroethanol [6] and correspond to the general notion of the typical structural changes accompanying the interaction between linear cationic AMP and the lipid bilayer. Many AMPs have a disordered structure in water and form an amphipatic α-helix upon binding with lipids (see reviews [14–16]).

Thus, the formation of the α-helix in complex with lipids, which is typical of Ltc1-K, as well as its size, is retained after the excision of one, two, or three N-terminal amino acid residues. The N-terminal of Ltc1-K is apparently a part of the region with the disordered structure, providing a gain in free energy during peptide binding with zwitterionic lipids due to its conformational mobility. This assumption is indirectly supported by the structure of Ltc1-K in complex with negatively charged sodium dodecyl sulphate micelles, determined by NMR [7]. The data obtained allow us to eliminate conformational rearrangement from the list of the possible reasons behind the lower cytotoxic and haemolytic activity of (-3)Ltc1-K in comparison with Ltc1-K.

**Comparative analysis of interaction of Ltc1-K and (-3)Ltc1-K with erythrocytes**

**Table 3:** Contribution of the α-helix conformation to the structure of Ltc1-K and its derivatives, based on the data of CD spectroscopy

Peptide	Phosphate buffered saline		LMPC micelles	
	%*	a.a.r.**	%*	a.a.r.**
Ltc1-K	17	4	59	15
(-1)Ltc1-K	14	4	67	17
(-2)Ltc1-K	15	4	72	17
(-3)Ltc1-K	16	4	65	15

* The proportion of the α-helix in the molecule structure.

** The number of amino acid residues involved in the formation of the α-helix.

The search for the possible reasons that determine the differences in the activity of Ltc1-K and (-3)Ltc1-K was taken further to the cell level. The features of haemolysis were compared at equi-effective concentrations of Ltc1-K and (-3)Ltc1-K (4 and 30 µМ, respectively), which were selected based on the concentration dependences of the haemolytic effect ( *Fig. 1* ). The difference in peptide concentration in the medium almost by a factor of 8 compensated for the differences between Ltc1-K and (-3)Ltc1-K in terms of affinity towards zwitterionic lipids ( *Table 2* ) and theoretically provided the same amount of Ltc1-K and (-3)Ltc1-K bound to the cell membrane.

**Fig. 4:**
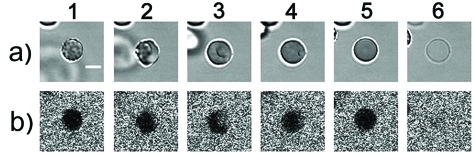
A sequence of Ltc1-K-induced erythrocyte transformations: echinocyte (1), echinocyte-stomatocyte transition (2), stomatocyte (3), stomatocyte-spherocyte transition (4), spherocyte (5), ghost (6). Row (a) is the transmitted light images of a cell. Row (b) is the confocal fluorescence images showing the distribution of CF around and inside erythrocyte. The black area in the cell region indicates that an erythrocyte membrane is intact, and CF is unable to penetrate into cytoplasm. Bar is 5 µm.

The interactions between peptides and the erythrocyte membrane were analyzed in real time by LSCM with the synchronous registration of cell images in the transmitted light (in order to assess the erythrocyte morphology) and fluorescence images characterizing membrane permeability in terms of the penetration of CF into the cytoplasm ( *Fig. 4* ). The ability of LCSM to register fluorescence only from a thin layer near the focus of the objective and to eliminate the signals from the layers located above and below is critical in order to obtain contrast images of the distribution of CF around and inside the cells.

**Fig. 5:**
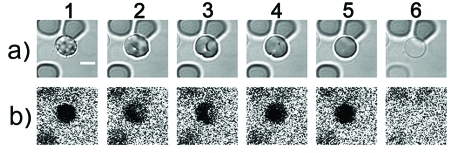
A sequence of (-3)Ltc1-K-induced erythrocyte transformations: echinocyte (1), echinocyte-stomatocyte transition (2), stomatocyte (3), stomatocyte-spherocyte transition (4), spherocyte (5), ghost (6). Row (a) is the transmitted light images of a cell. Row (b) is the confocal fluorescence images showing the distribution of CF around and inside erythrocyte. The black area in the cell region indicates that an erythrocyte membrane is intact, and CF is unable to penetrate into the cytoplasm. Bar is 5 µm.

The exposure to Ltc1-K and (-3)Ltc1-K results in the equally complete release of haemoglobin from some erythrocytes and converting them into ghosts, whereas the proportion of lysed erythrocytes increases with a rising peptide concentration in the medium. The haemolysis process occurs very inhomogeneously: a number of erythrocytes are not subjected to any noticeable changes, whereas morphological changes emerge in some erythrocytes with a different delay after the peptide is introduced, which results in ghost formation. The differences in cell response are not connected with the local concentration gradients of the peptides introduced. The high inhomogeneity factor of peptide distribution in the medium was eliminated by means of thorough and rapid stirring of equal volumes of the peptide and cell suspensions. The exposure to Ltc1-K and (-3)Ltc1-K results in an equal sequence of erythrocyte transformations ( *Figs. 4, 5* ): discocyte–echinocyte– stomatocyte–spherocyte–ghost. The lifetime of an echinocyte varies over a wide range (from several minutes to an hour) and is the limiting stage of haemolysis kinetics. The average duration of the stomatocyte stage is several minutes. Spherocyte is the shortest living state. It lasts no more than 10 s. No reliable differences in the duration of the separate stages of erythrocyte transformation under the action of both peptides were observed. The increase in peptide concentration in the medium primarily resulted in a decrease in the average lifetime of echinocytes.

**Fig. 6:**
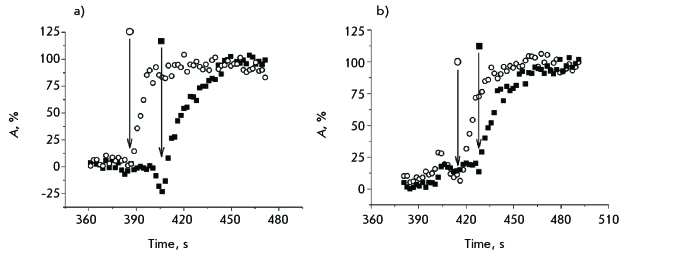
Ltc1-K- (a) and (-3)Ltc1-K (b)-induced formation of membrane pores and influx of CF in erythrocytes. Typical examples are presented for two particular cells (○, ■). Abscissa is time after addition of the peptide and CF to cells. Ordinate is the extent of CF influx inside a cell (in percentages) as calculated with equation (3). Delay between peptide addition and beginning of haemolysis is the property of particular cells and is mainly defined by echinocyte-stage lifetime. A vertical arrow indicates the moment when haemoglobin release from the examined cell as recognized in transmitted light images of the cell begins.

Only at the stage when spherocyte was converted into the ghost did the erythrocyte membrane become permeable to the low-molecular-weight CF marker; a factor which is common to both peptides ( *Figs. 4–6* ). The influx of CF in cytoplasm and the beginning of haemoglobin release from a spherocyte ( *Fig. 6* ) are detected simultaneously. This implies the formation of large membrane defects without the indiscernible under our experimental conditions intermediate study of the formation of small lipid-peptide pores that would be permeable to CF (hydrodynamic diameter of the molecule of ~0.5 nm), but impermeable to haemoglobin (hydrodynamic diameter of 6.3 nm).

**Fig. 7:**
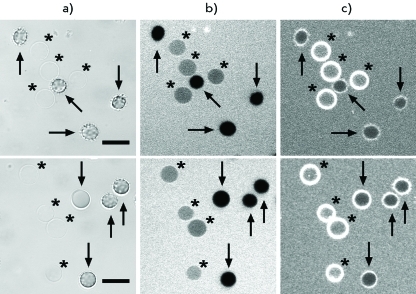
LSCM analysis of FD500 ability to penetrate through the ghost membrane pores produced by Ltc1-K (upper row) and (-3)Ltc1-K (lower row). FD500 and membrane marker FM1-43 were added to the erythrocytes pretreated with the peptide. Column (a) is the transmitted light images of the cells. Column (b) is the confocal fluorescent images showing the distribution of FD500 around and inside the cells. Column (c) is the confocal fluorescence images showing the distribution of FM1-43 at an erythrocyte membrane. Asterisks mark ghosts being restrictedly permeable to FD500. Arrows mark erythrocytes being impermeable to FD500. Bar – 10 µm.

**Fig. 8:**
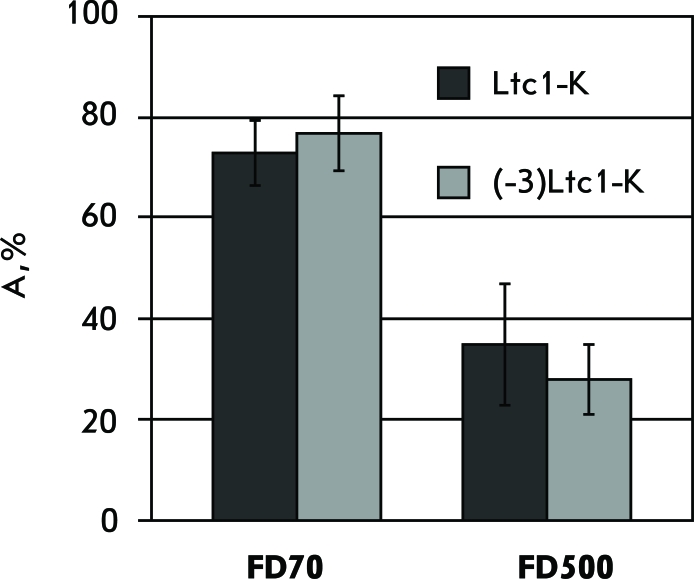
Extent of influx (A) of the membrane permeability markers FD70 and FD500 into the ghosts formed as a result of Ltc1-K and (-3)Ltc1-K action on erythrocytes. Averaged data for 100-150 cells are presented.

We found out that the membrane of the ghosts that remained under the action of Ltc1-K and (-3)Ltc1-K retains its permeability to CF, as opposed to the membrane of the ghosts emerging after osmotic haemolysis of erythrocytes caused by the replacement of the medium by distilled water (data not shown). Therefore, Ltc1-K and (-3)Ltc1-K form stable lipid-peptide pores in the erythrocyte membrane. The average size of these pores was estimated on the basis of the ability of fluorescein-labelled dextrans of various sizes to penetrate through the pores inside the ghosts. It was revealed that dextrans with a size of 40 kDa and lower can easily penetrate into the ghosts to rapidly balance the extra- and intracellular concentrations when added to the medium with lysed erythrocytes. Meanwhile, the diffusion of dextrans with a size of 70 and 500 kDa through pores into the ghosts is impeded ( *Figs. 7, 8* ). The extents of influx are approximately 70% and less than 40% for FD70 and FD500, respectively ( *Fig. 8* ). No reliable difference in the penetration of FD70 and FD500 into the ghosts formed under the action of Ltc1-K and (-3)Ltc1-K was detected ( *Fig. 8* ). Considering the hydrodynamic diameters of FD70 and FD500 (13 and 32 nm, respectively [17]), one may conclude that Ltc1-K and (-3)Ltc1-K form pores with a diameter of approximately 30 nm on the erythrocyte membrane.

Thus, the excision of the N-terminal fragment has no effect on the character, sequence, and duration of the stages of erythrocyte transformation or the features of formation of the pore size in the membrane in the haemolysis process. This attests to the fact that the mechanism and features of interaction of Ltc1-К and (-3)Ltc1-K with erythrocytes are equal. 

The model of interaction of Ltc1-К and (-3)Ltc1-K with erythrocytes can be based on the coupled bilayer theory [18–21]. In this theory, an asymmetric plasma membrane is regarded as two protein–lipid layers that are capable of performing a relatively independent adaptive deformation without losing the contact between them. The coupled bilayer theory has frequently been used to interpret the formation of echinocytes and stomatocytes under the action of membrane-active agents, including AMPs [18, 22, 23]. According to this theory, echinocyte formation is conditioned by the accumulation of the actuating agent in the external layer of the membrane. The interactions with any agent, which stretch the external layer with respect to the internal layer, result in the formation of “spicules” – protrusions on the membrane, which are a characteristic feature of echinocytes. In turn, the formation of stomatocytes is induced by the accumulation of the agent in the internal layer and its deformation, which ends by the transformation of a discocyte into a spheroid with membrane cavity.

The interpretation of erythrocyte transformations under the action of Ltc1-K and (-3)Ltc1-K using the coupled bilayer theory is complicated by the fact that the scenarios of the formation of echinocyte and stomatocyte are considered to be alternative. Assuming that the translocation of the agent from the external layer to the internal one is accompanied by echinocyte transformation into a discocyte, the interactions of Ltc1-K and (-3)Ltc1-K with erythrocytes can be explained as follows: the transition of a discocyte into an echinocyte is initiated by the initial adsorption of a peptide by the erythrocyte surface. The accumulation of a peptide bound on the external side of the membrane results in the reorganization of lipid–peptide complexes and creates the conditions for peptide translocation to the internal surface of the plasma membrane, resulting in the transformation of an echinocyte into a stomatocyte. It should be emphasized that the hypothetical translocation of a peptide is not accompanied by the formation of structured defects in the bilayer, since the membranes of both the echinocyte and stomatocyte are impermeable to CF ( *Figs. 4, 5* ). The stomatocyte–spherocyte transition probably corresponds to a considerable accumulation of a cationic peptide in the internal membrane layer that is enriched in negatively charged phospholipids, and to the formation of a critical tension in both membrane layers. The process is terminated by the formation of lipid–peptide pores, which reduce the membrane tension and create the conditions for haemoglobin release and ghost formation.

This model of interactions of Ltc1-K and (-3)Ltc1-K with erythrocytes agrees with the lifetimes of the separate stages of erythrocyte transformation recorded in the experiment. Long erythrocyte lifetimes represent the continuous process of gradual accumulation of the peptide on the external side of the membrane. This process is accelerated with increasing peptide concentration in the medium. An increase in the amount of the peptide bound to the membrane to a critical level jumpwise increases the probability of peptide translocation to the internal layer of the membrane and considerably stabilizes the membrane, which is manifested in the acceleration of the subsequent processes resulting in haemolysis. 

All the data obtained indicate that the binding of a peptide to the external layer of the erythrocyte membrane, with zwitterionic phospholipids dominated in its composition, and the accumulation of a critical amount of peptides on the membrane trigger the haemolysis process. The excision of the N-terminal fragment of Ltc1-K abruptly decreases the ability of the peptide to be bound to the membrane, thus protecting erythrocytes against haemolysis. An increase in the concentration of (-3)Ltc1-K in the medium compensates for the deficiency in bound peptide, all subsequent haemolysis stages with the participation of (-3)Ltc1-K occurring in the same manner as for the initial peptide Ltc1-K. 

It can be assumed that the mechanism of the decrease in the toxicity of peptides that was proposed for a number of linear cationic AMPs and demonstrated by the example of latarcins Ltc2а and Ltc5 by excising their N-terminal fragments [5] is similar to the mechanism ascertained for Ltc1-K. The N-terminal fragment seems to be responsible for the efficacy of peptide binding to the zwitterionic plasma membrane of eukaryotic cells when AMPs are organized according to the scheme “non-ordered hydrophobic N-terminal fragment + amphipatic α-helix.” Provided that the affinity of the N-terminal fragment of AMPs towards eukaryotic membranes is higher than that towards bacterial membranes, safety in the use of AMPs as an antibacterial agent can be improved by excising the N-terminal amino acids.

## CONCLUSIONS 

The excision of three N-terminal amino acid residues in Ltc1-К considerably reduces the haemolytic activity of AMPs, thus increasing the selectivity of its antibacterial action. 

The major reason for the effect observed consists in a multifold decrease in peptide affinity towards the plasma membrane of eukaryotic cells enriched in zwitterionic phospholipids. 

The haemolysis caused by the AMP of Ltc1-K and its derivative (-3)Ltc1-K is a sophisticated multistage process. The binding of peptides to the zwitterionic membrane of erythrocytes is accompanied by their transition into the α-helix conformation. By stabilizing the membrane, the bound peptides transform an erythrocyte from a discocyte into a echinocyte, followed by a transformation into a stomatocyte and a spherocyte. When the sufficient amount of peptide necessary to form pores accumulates on the membrane, spherocyte undergoes haemolysis, yielding stable lipid-peptide pores approximately 30 nm in diameter. The role of the N-terminal fragment of Ltc1-K consists in the enhancement of peptide binding to the erythrocyte membrane. The N-terminal fragment has no effect on the features of the subsequent stages of peptide interaction with erythrocyte, which results in haemolysis. 
